# Ultrasonic Vibration-Assisted Micro-Electrical Discharge Machining Characteristics and Parameter Optimization of C/SiC Composites

**DOI:** 10.3390/mi16111257

**Published:** 2025-11-04

**Authors:** Peng Yu, Lize Wang, Yongcheng Gao, Qiang Li, Yiquan Li

**Affiliations:** Ministry of Education Key Laboratory for Cross-Scale Micro and Nano Manufacturing, Changchun University of Science and Technology, Changchun 130022, China; 15665938736@163.com (L.W.); 15704301002@163.com (Y.G.); 13620788904@163.com (Q.L.)

**Keywords:** ultrasonic vibration, micro-EDM, C/SiC composites, removal mechanism, response surface methodology

## Abstract

Carbon-fiber-reinforced silicon carbide (C/SiC) composite materials, as a kind of composite material with the characteristics of ceramics, have the following characteristics: high strength, high stiffness, low density, high temperature resistance, and high corrosion resistance. These characteristics make them widely applicable in aerospace, defense, automotive, and other high-performance industries. However, because of their anisotropy and inherent brittleness, ceramic-based composites are still difficult to process using conventional processing techniques. In this study, a technique of ultrasonic vibration-assisted micro-electrical discharge machining (micro-EDM) for the precise machining of two-dimensional (2D) C/SiC composites was proposed. The research mainly focused on an investigation of the material removal mechanism of C/SiC composites under ultrasonic vibration-assisted micro-EDM conditions. The erosion process was found to involve melting and vaporization of the SiC matrix, whereas the carbon fibers were removed by fragmentation and localized melting. In order to assess the effects of various process parameters on the material removal rate (MRR), single-factor experiments were performed initially. Afterwards, response surface methodology was used to optimize the MRR of C/SiC composites during ultrasonic vibration-assisted micro-EDM. A Plackett–Burman (PB) design was used to determine the parameters that have a significant effect on MRR. Based on these results, the optimum parameter range was obtained using the method of steepest ascent. Finally, a Box–Behnken design was used to determine the best machining parameters for improved performance.

## 1. Introduction

Carbon-fiber-reinforced silicon carbide (C/SiC) composite materials have low density, high strength, good wear resistance, and high chemical corrosion resistance. They also have excellent mechanical properties and structural stability at temperatures above 2000 °C [1]. In recent years, C/SiC composites have shown a wide range of application prospects in aerospace, energy, and high-temperature thermal protection systems. These materials are typical fiber-reinforced composites and comprise a matrix of silicon carbide (SiC) and a reinforcing structure of carbon fiber. The two-dimensional (2D) C/SiC composite is a typical type of composite with an interwoven layer structure and no fibers in the Z-axis direction. However, during the machining process, it tends to suffer from defects like delamination and burr formation due to interlayer stresses [2], which are obstacles to precision manufacturing. Due to their high hardness, conventional milling tools suffer severe wear when used for C/SiC composites, while laser machining is often affected by unstable forming quality [3]. Electrical discharge machining (EDM), which removes material through high-temperature erosion due to electrical discharges between electrodes, does not involve direct mechanical contact and stress, so the tool wear is less than that in traditional machining. Moreover, the high electrical conductivity that the carbon fibers give to the composite makes EDM a very feasible and effective technique to machine C/SiC composites [4].

Although the EDM of ceramic matrix composites has received much academic attention, there is still little research on C/SiC composites, and this research is mainly concerned with whether the process is feasible. Zhang et al. (2018) used electrodes of different dimensions to machine C/SiC composites and proved that micro-EDM is appropriate for processing such difficult-to-machine materials with a machining accuracy of <0.5 mm [5]. Guu et al. (2001) were one of the first to use various micro-EDM parameters on ceramic-based composites, and they found that delamination, recast layer thickness, and surface roughness were all positively correlated with input power [6]. Yue et al. (2020) studied the discharge and etching mechanism of Cf/SiC composites via micro-EDM, and found that thermal stress is an important factor to ensure efficient material removal [7]. Similarly, He et al. (2019) investigated the effect of machining parameters and fiber orientation on machining speed and surface roughness using an orthogonal experimental design for wire EDM of C/SiC composites [8]. In general, these studies are mainly concerned with the feasibility and parameter optimization of EDM technology, whereas the studies that have investigated the detailed material removal mechanism and the potential advantages of energy-field-assisted machining for C/SiC composites are relatively limited.

For conventional EDM of ceramic materials, the material removal rate (MRR) is generally very low and is around 0.04 mm^3^/min [9]. In the case of traditional EDM, material removal is mostly a result of spalling due to thermal shock. The alternating thermal loads can easily cause residual stresses in the ceramic, which will cause surface microcracks, edge collapse, and other defects [10]. In the discharge process between the tool electrode and the workpiece, localized melting and vaporization take place at very high temperatures. However, the resulting debris is often hard to remove, which has adverse effects on the machining stability and efficiency. To solve these problems, ultrasonic vibration has been introduced to help the process of micro-EDM. Wang et al. investigated the cavitation effect induced by ultrasonic vibration in EDM and found that the collapse of cavitation bubbles produces impact forces that improve the surface quality and decrease surface roughness [11]. Similarly, Zhang et al. reported that the introduction of ultrasonic vibration in EDM enhances the velocity gradient of the flow field by controlling the vibration amplitude for improving the fluid conditions of the discharge gap [12]. These results support the conclusion that ultrasonic vibration has a significant effect on the machining process. The cavitation effect creates many microscopic bubbles that, when collapsing, create powerful shock waves that have the power to fragment and disperse molten material, aiding in the removal of the debris. In addition, the pumping effect also promotes the timely evacuation of molten material, which prevents resolidification on the machined surface. Considering that traditional EDM generally exerts flaws while machining C/SiC composites reinforced by carbon fibers, this study proposes the use of ultrasonic vibration-assisted micro-EDM in fabricating micro-holes in C/SiC materials and aims to improve the MRR using optimized process parameters.

Due to the special microstructure of C/SiC, many scholars at home and abroad have studied ultrasonic vibration-assisted micro-EDM. Xing et al. used ultrasonic vibration-assisted micro-EDM technology on titanium alloys and obtained a 2.4 times increase in the MRR, a decrease in the relative tool wear rate of 65.8%, a decrease in taper angle of 73%, and a decrease in overcut of 32% [13]. Wang et al. created a microscopic material removal model for single-pulse EDM using simulation, which showed that ultrasonic vibration reduces carbon deposition area and surface finish by reducing surface roughness [14]. These results together show the positive effects of ultrasonic vibration on the performance of EDM.

In this study, ultrasonic vibration was added to the EDM of C/SiC composites to study the effect of ultrasonic vibration on the machining behavior and material removal characteristics. A mathematical model was derived to describe the relationship between process parameters and MRR and allow an assessment of the relative importance of individual variables and the determination of optimal process parameters for ultrasonic vibration-assisted EDM of C/SiC composites.

## 2. Experimental Platform and Method

### 2.1. Experimental Platform

**Figure 1:**
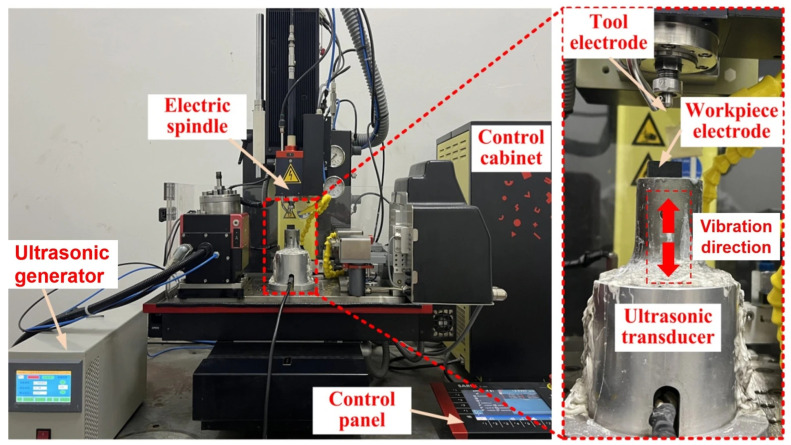
Micro-EDM machine tool.

The experimental setup used in this study was a micro-EDM machine tool with a custom-designed piezoelectric ceramic ultrasonic transducer, as shown in Figure 1. The machining system used was a five-axis linkage ultra-precision micro-EDM machine (Model: SM-100HPM). The travel ranges of the Z-, X-, and Y-axis stages were 250, 150, and 150 mm, respectively. The positioning accuracy of the machine was ±2 μm with a resolution of 0.1 μm. The ultrasonic transducer produced high-frequency vibrations at 20 kHz, with adjustable amplitudes of 3 μm to 12 μm driven by an ultrasonic generator.

The main components of the system were the control cabinet, control panel, electric spindle, ultrasonic generator, ultrasonic transducer, and tool electrode. The control cabinet was responsible for the powering of the equipment, whereas the control panel allowed for positioning of the electric spindle to the desired machining location and execution of machining programs. The ultrasonic system, provided by Hangzhou Chenggong Ultrasound Company (Hangzhou, China), had a power of 1500 W and a frequency of 20 kHz. The ultrasonic generator offered an amplitude setting for the transducer. As shown by the red dashed outline in Figure 1, the ultrasonic transducer was placed directly above the workpiece during machining, and its vibration direction was parallel to the axial direction of the tool electrode. During processing, the periodic axial vibration of the transducer caused both cavitation and pumping effects in the working fluid, which improved debris removal and improved the overall machining performance.

### 2.2. Experimental Materials

**Figure 2:**
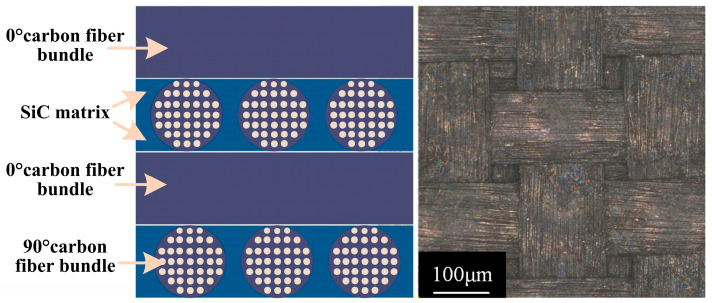
Structural diagram and microscopic image of 2D C/SiC composites.

The work material in this experiment was a 2D C/SiC composite. Compared with three-dimensional (3D) weaving techniques, the 2D configuration makes the carbon fiber arrangement pattern relatively simple [15,16]. As shown in Figure 2, the 2D C/SiC composite is woven with 0deg and 90deg fiber bundles and the fiber spaces are filled with a SiC matrix. This structure lacks any longitudinal connecting fibers. The tool electrode was a tungsten–cobalt alloy rod with a diameter of 0.21 mm provided by the Swiss brand SARIX (Model: SE03).

The addition of carbon fibers changes the distribution of the SiC matrix and thus leads to anisotropic properties. As a result, the physical properties of C/SiC composites are not specific, but fall within a range. In this composite, carbon fiber is the reinforcing phase and the main conductive phase, and SiC has a relatively low electrical conductivity. The carbon fibers used were PAN-based, with an electrical conductivity of 65,000 S/m. Each fiber bundle consisted of about 12,000 filaments with a diameter of 7.0 µm, a density of 1760 kg/m^3^, a transverse tensile modulus of 15 GPa, and a longitudinal tensile modulus of 230 GPa. The SiC matrix had an electrical conductivity of 3.2 S/m, a density of 1200 kg/m^3^, and a tensile modulus of 3.5 GPa. Therefore, the overall electrical conductivity of the C/SiC composite is between carbon fiber and SiC. The physical properties of C/SiC composites are summarized in Table 1.

### 2.3. Experimental Method

The machining capability of C/SiC composites under micro-EDM conditions was analyzed in terms of the MRR. The MRR is the amount of material removed from the workpiece per unit time and is given by the following equation:

(1) $$MRR\;=\;\frac{V}{t}\left({\mathrm{mm}}^{3}/s\right)$$

In this equation, *V* is the volume of material removed from the workpiece, and *t* is the total machining time.

In the calculation of MRR, the machining time is the sum of time from the moment when the electrode touches the workpiece to the moment when the machining is finished, which is automatically measured by the control system of the machine tool. The volume of material removed, *V*, is calculated using the following equation:

(2) $$V=\pi \times {\left(\frac{D_{avg}}{2}\right)}^{2}\times H\;\left({mm}^{3}\right)$$

In the equation, *D_avg_* is the average diameter, and *H* is the aperture depth.

The microstructure and size of the machined workpiece were analyzed by using high-accuracy observation equipment. A confocal laser scanning microscope (CLSM) was used to analyze the 3D morphology of the workpiece surface, which can visualize the surface texture, peak–valley variation, and topographical features. This method was used to visualize the 3D morphology of the machined surface. The fine surface features of the workpiece were observed and the pore dimensions were measured with a scanning electron microscope (SEM), which has a nanometer-scale resolution. In this study, the surface morphology of the micro-holes along the inner and outer edges and the pore sizes were characterized using SEM.

In this study, ultrasonic vibration-assisted micro-EDM was adopted, in which cavitation and pumping effects induced by ultrasonic vibration contribute to the increase in MRR for machining C/SiC composites. The mathematical expression of the coupling relationship between ultrasonic vibration and the discharge gap is given in equation:

(3) $$d\left(t\right)\;=\;d_{0}\pm Asin(2\pi ft)$$

In this equation, *d*_0_ is the initial electrode-to-workpiece discharge gap; *A* is the amplitude of vibration; and *f* is the frequency of vibration. The function *sin*(2*πft*) is used to describe the periodic time-varying nature of vibration. The dynamic evolution of the discharge gap between the tool electrode and the C/SiC composite workpiece is expressed by this term. The calculated values are related to the instantaneous discharge gap *d(t)*, which is the instantaneous separation between the tool and workpiece electrodes at any instant of time.

**Figure 3:**
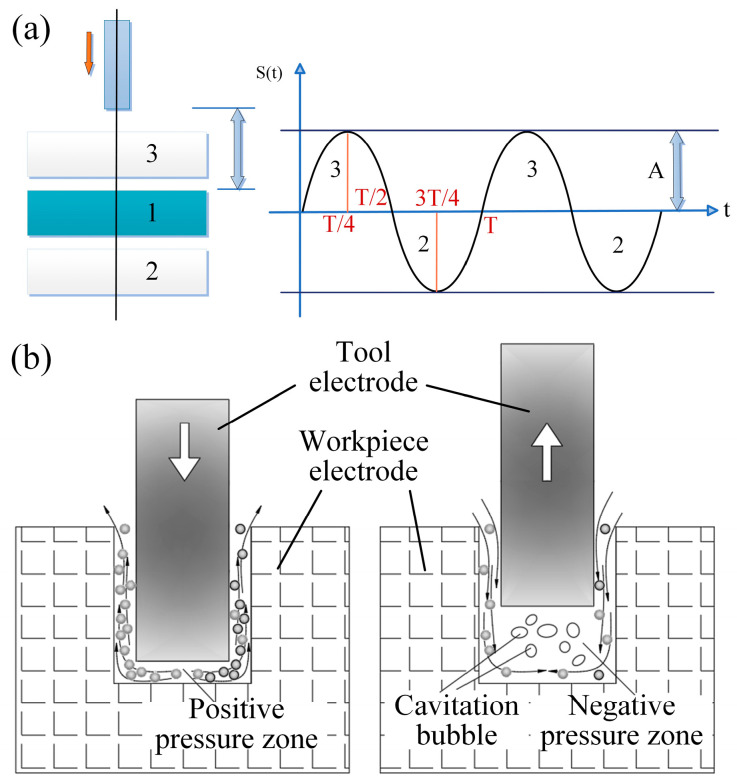
Schematic diagram of ultrasonic vibration system and its principle of action in EDM. (**a**) Schematic diagram of ultrasonic vibration model; (**b**) principle diagram of ultrasonic vibration action.

Figure 3a is a schematic of the ultrasonic vibration model, where the tool electrode vibrates at high frequency with a period T under ultrasonic excitation. S(T) represents the displacement of the electrode with time during ultrasonic vibration. The electrode motion is periodic: downward motion is taken as positive and upward motion as negative. The maximum and minimum values of S(T) are the extreme positions of the electrode in the positive and negative directions, respectively, with respect to its initial position. At T/2 and T, the instantaneous displacement S(T) goes back to zero. This model shows the dynamic motion behavior of the electrode under ultrasonic vibration. These oscillations cause cavitation and pumping effects in the discharge gap, as shown in Figure 3b. As the electrode vibrates, it produces high-frequency elastic waves in the working fluid. When the local pressure falls below the saturated vapor pressure of the fluid, transient negative-pressure regions form, which results in the formation of microscopic cavitation bubbles. Upon collapse, these bubbles release instantaneous impact forces that increase jet velocity within the discharge channel, effectively detaching debris from the bottom of the machined hole. This mechanism ensures the debris is not accumulated, and thus both the machining quality and efficiency are improved. The pumping effect is achieved by the high-frequency reciprocating motion of the electrode, which compresses and releases the working fluid in the discharge gap alternately. When the electrode is moved away from the workpiece, the volume of the gap is increased, creating a localized low-pressure area which draws fresh working fluid quickly into the gap. Conversely, as the electrode gets closer to the workpiece, the volume of the gap reduces and creates a localized high-pressure zone, which forces debris-laden fluid outward. This process is efficient in terms of erosion debris removal and its accumulation prevention, ensuring uninterrupted fluid renewal and restoring the insulating strength of the dielectric. As a result, abnormal discharges are minimized, the machining process is stabilized, and the occurrence of low-resistance conduction states in which the current flows at low voltage and high current, causing the energy dispersion without effective erosion, is effectively suppressed.

The parameter ranges were established by conducting extensive preliminary experiments, in which a large number of values for 5 key process parameters were tested, including peak current, peak voltage, pulse frequency, pulse width, and vibration amplitude. The peak current was varied from 10 to 50 Index, the peak voltage from 20 to 180 V, the pulse frequency from 40 to 200 kHz, the pulse width from 1 to 9 µs, and the vibration amplitude from 0 to 8 µm. From these experiments, characteristic trends were noticed within the ranges of the parameters, as summarized in Table 2.

In order to further clarify the effect of a single parameter on the EDM performance of C/SiC composites, a series of single-factor experiments were carried out. The parameter values adopted for these experiments are given in Table 2.

In micro-EDM, the working fluid is used mainly to wash the chips and the heat produced during machining. The working fluid chosen in this study was EDM oil, which was chosen for its high insulation strength, large dielectric constant, and high viscosity. In this paper, the machining liquid is referred to as the working fluid uniformly. The physical properties of working fluid are given in Table 3.

## 3. Experimental Results and Analysis

### 3.1. Effect of Ultrasonic Vibration on Machining

**Figure 4:**
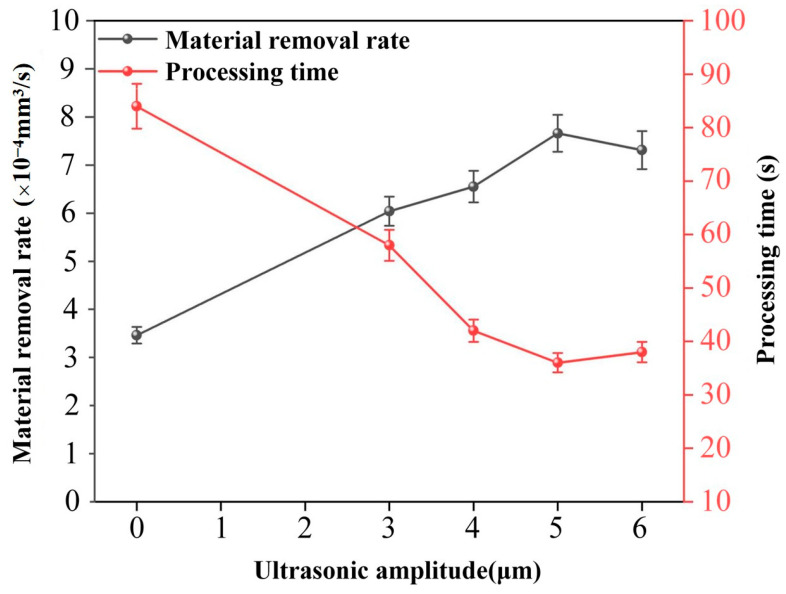
Effect of ultrasonic amplitude on MRR and processing time.

Figure 4 shows the effect of ultrasonic amplitude on the MRR and processing time under the conditions of a peak current of 30 Index, peak voltage of 100 V, pulse frequency of 120 kHz, and pulse width of 5 µs. As the ultrasonic amplitude increases, the MRR increases, while the processing time decreases. When the amplitude is 5um, the MRR reaches its maximum value, and the processing time reaches its minimum. Beyond this amplitude, the MRR starts to decrease, and the processing time correspondingly increases.

The ultrasonic vibration of the electrode causes the pressure of the machining zone to alternate. With increasing amplitude, the intensity of this alternating pressure becomes stronger. The resulting impact pressure makes it easier for erosion debris to detach from the surface of the workpiece. Simultaneously, the increased suction effect produced by the greater amplitude allows for speedy inflow of fresh working fluid into the discharge gap. This constant renewal of the fluid creates a cyclic process of efficient discharge, rapid debris removal, and re-discharge of the fluid, which improves the MRR and shortens the overall machining time.

When the ultrasonic amplitude is greater than 5 µm, the MRR starts to decrease. Although the cavitation effect still helps to remove the debris, an excessively large amplitude creates many oversized cavitation bubbles. Because these bubbles are non-conductive, they block the development of discharge channels. Consequently, stable discharges are not possible because the bubbles separate the electrode from the working fluid, and the number of effective discharges is reduced. Moreover, although an increased amplitude improves the alternating pressure in the discharge gap, beyond a critical value it causes rapid and irregular pressure fluctuations. These fluctuations cause disordered fluid flow, such as eddies and backflows, which make it difficult to remove the debris effectively. Instead of being expelled, debris-laden fluid may re-enter the machining zone and cause reaccumulation and degraded discharge stability. As a result, the MRR is reduced and the processing time is increased.

**Figure 5:**
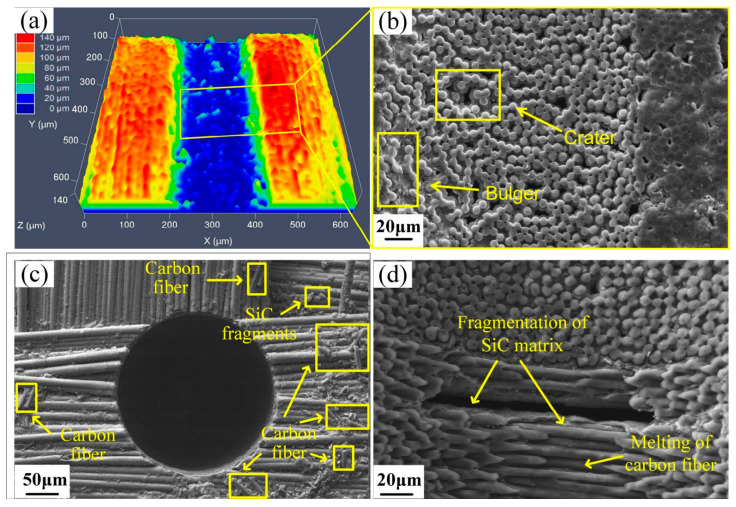
CLSM and SEM images of the C/SiC composite surface after EDM. (**a**) 3D morphology of the micro-hole. (**b**) Morphology of the micro-hole wall obtained using SEM. (**c**) Surface morphology at the micro-hole entrance. (**d**) The hole wall morphology with material removal features.

Figure 5 shows the CLSM and SEM images of the surface of the C/SiC composite after EDM. Figure 5a presents the 3D morphology of the micro-hole created via EDM, where the blue region represents the machined area. The uneven color depth shows great height variations, and local pits and protrusions may be present. Figure 5b shows the SEM morphology of the micro-hole, and the cross-sections of carbon fibers and SiC matrix between them can be clearly seen. These features are in agreement with the 3D morphology, both revealing pits and protrusions. The observed morphology proves that during EDM of C/SiC composites, the removal of the SiC matrix leaves protruding carbon fibers, indicating that the erosion rate and volume of the SiC matrix are higher than the carbon fibers.

Figure 5c presents the morphology of the micro-hole entrance after ultrasonic vibration excitation, in which the yellow areas are carbon fibers and SiC debris. The presence of these residues around the hole entrance clearly indicates that the EDM process of C/SiC composites is accompanied by the melting of the SiC matrix and cracking of the carbon fibers. Melting and fragmentation of the SiC matrix reveal the carbon fibers, which, under the impact of the explosive force of sparks, are prone to breaking and generating debris. Ultrasonic vibration helps the working fluid pump the fragments out of the hole quickly, which are concentrated around the entrance. From the carbon fiber fracture surface in Figure 5d, it can be seen that the fiber removal also includes melting, because the fracture surface has traces of melting and not just mechanical fracturing. This result reveals that the melting and vaporization of the SiC matrix, as well as the combined fracture and melting of carbon fibers, are the primary material removal mechanisms in ultrasonic vibration-assisted micro-EDM of C/SiC composites.

**Figure 6:**
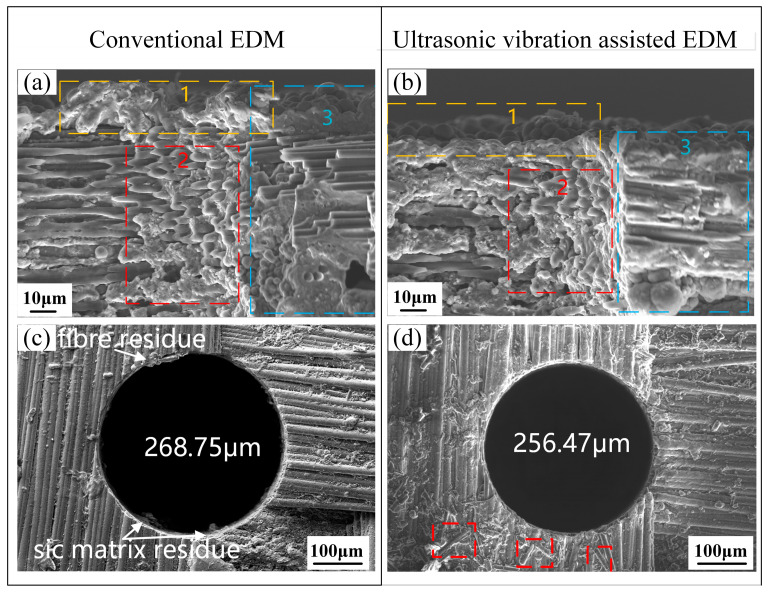
Comparison of the recast layer and hole diameter of C/SiC composites processed using conventional EDM and ultrasonic vibration-assisted EDM. (**a**) Recast layer after conventional EDM. (**b**) Recast layer after ultrasound-assisted EDM with an amplitude of 5 μm. (**c**) Hole diameter after conventional EDM. (**d**) Hole diameter after ultrasonic vibration-assisted EDM with an amplitude of 5 μm.

Figure 6 shows a comparison of the recast layer and hole diameter of C/SiC composites processed using conventional EDM and ultrasonic vibration-assisted EDM. In locations 1 and 2 of Figure 6a, the recast layer is relatively thick, unevenly shaped, rough, and unevenly distributed. When the hole depth is deep, the high temperature generated under discharge causes the rapid melting and partial vaporization of the workpiece surface. Because of the very short discharge time, a part of the molten material cannot be forced out of the discharge area in time and resolidifies on the surface, resulting in the recast layer seen in Figure 6a. In contrast, the recast layer in Figure 6b is much thinner and more homogeneous. This is due to the adoption of ultrasonic vibration, which generates a great number of microbubbles that collapse to generate intense shock waves due to cavitation. These shock waves break up and disperse molten material, which helps to remove erosion debris. At the same time, the pumping effect quickly removes the molten material from the discharge gap to avoid resolidification of the molten material on the machined surface. A large decrease in carbon fiber delamination is also observed in area 3 of Figure 6b due to the improved fluid flow and debris removal caused by ultrasonic vibration. The enhanced heat dissipation reduces thermal accumulation in the machining area and thermal stress caused by the difference in thermal expansion coefficient between carbon fibers and SiC matrix, thus reducing delamination.

Figure 6c shows the morphology of the micro-hole entrance after conventional EDM; the hole diameter was 268.75 µm. Figure 6d shows the hole entrance morphology obtained at an ultrasonic amplitude of 5 μm, with a smaller diameter of 256.47 µm. Residual fractured carbon fibers are shown in the red box regions of Figure 6d. After ultrasonic vibration is introduced, the carbon fibers broken by cavitation are washed out by the working fluid and are gathered around the hole entrance, which verifies that carbon fiber fracture is one of the main erosion mechanisms in the ultrasonic vibration-assisted micro-EDM of C/SiC composites. The hole diameter decreased by about 12 μm after ultrasonic vibration was added. The improved removal of debris in the discharge gap caused by cavitation and pumping effects eliminates unstable discharge channels that can focus energy on the hole wall and produce excessive erosion and hole enlargement.

### 3.2. Effect of Electrical Parameters on MRR

#### 3.2.1. The Effect of Peak Current on MRR

**Figure 7:**
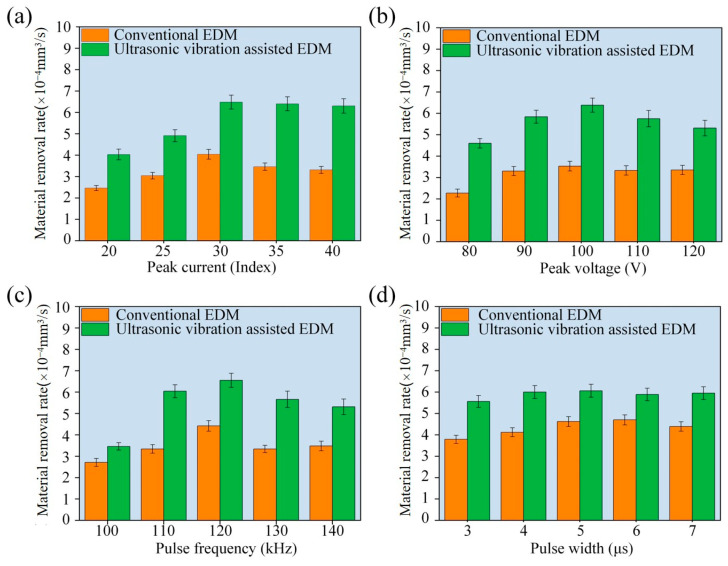
Effect of electrical parameters on MRR in conventional EDM and ultrasonic vibration-assisted EDM. (**a**) Peak current; (**b**) peak voltage; (**c**) pulse frequency; (**d**) pulse width.

Figure 7a shows the effect of peak current on MRR under the conditions of peak voltage of 100 V, pulse frequency of 120 kHz, pulse width of 5 µm, and ultrasonic amplitude of 5 µm. Both conventional EDM and ultrasonic vibration-assisted EDM have a characteristic rise–fall trend: the MRR increases with peak current up to a certain point and then gradually decreases. Notably, the ultrasonic-assisted process is more consistent in terms of MRR across the entire range. Here, Index represents a specific quantitative unit defined by the micro-EDM equipment manufacturer to describe the peak current. In this device, the actual peak current is equal to four times the Index value. The largest increase in MRR occurs as the current increases from 20 to 30 Index, after which it starts decreasing. This initial growth is due to the increase in the discharge energy released per pulse as the peak current increases.

The increased heat-affected zone leads to more extensive melting and vaporization of the material, and thus an improved MRR. When the peak current becomes greater than 30 Index, the MRR starts to decrease. Excessively high current produces large amounts of erosion products and debris, which cannot be removed quickly enough to allow energy transfer to the machining surface and reduces the MRR. For conventional EDM, this reduction is sharp, while for ultrasonic vibration-assisted EDM, the reduction is more gradual. This difference comes from the fact that ultrasonic vibration improves working fluid circulation and speeds up the removal of debris, which helps to overcome the negative effects of debris accumulation. In summary, the optimum peak current for the maximum MRR in ultrasonic vibration-assisted micro-EDM of C/SiC composites is about 30 Index.

#### 3.2.2. The Effect of Peak Voltage on the MRR

Figure 7b shows the effect of peak voltage on the MRR with a peak current of 30 Index, pulse frequency of 120 kHz, pulse width of 5 µs, and ultrasonic amplitude of 5 µm. The MRR first increases and then decreases with the increase in peak voltage. When the peak voltage is between 80 and 100 V, the MRR increases significantly and reaches a maximum value; when the peak voltage is greater than 100 V, the MRR decreases. The initial increase in MRR can be explained by the increase in the electric field inside the discharge channel and the corresponding increase in plasma temperature at high voltages. The extra heat transferred to the surface of the C/SiC workpiece enhances the melting and vaporization of the material. In addition, due to the increase in the voltage, the discharge channel is stabilized, which improves the machining stability and increases the MRR. However, as the applied voltage increases further above 100 V, excessive deposition of debris in the discharge gap causes serious local variations in electric field strength. As a result, the discharge breakdown takes place preferentially at these localized areas of high energy, rather than in the desired machining zone, resulting in scattered and unstable discharges. The dispersion of discharge energy shortens the effective machining time and decreases the energy utilization efficiency, which eventually results in a decrease in the MRR. In conclusion, the best peak voltage for the maximum MRR in ultrasonic vibration-assisted micro-EDM of C/SiC composites is about 100 V.

#### 3.2.3. The Effect of Pulse Frequency on the MRR

Figure 7c shows the influence of pulse frequency on the MRR with the parameters of a peak current of 30 Index, peak voltage of 100 V, pulse width of 5 µm, and ultrasonic amplitude of 5 µm. As shown in the figure, the MRR increases significantly when the frequency of the pulse is in the range of 100 to 120 kHz, but starts to decrease when the frequency is higher than 120 kHz. The initial increase in the MRR is attributed to the increase in the number of discharges per unit time with the increase in pulse frequency. A higher discharge frequency increases the rate of energy accumulation in each discharge channel, which results in a faster heat transfer to the C/SiC workpiece surface. This helps to increase the melting and vaporization of the material, which helps to improve the MRR. However, when the pulse frequency exceeds 120 kHz, due to the extremely short interval between pulses, the erosion debris cannot be taken away by the working fluid effectively, and the debris will accumulate in the discharge gap. Furthermore, the high-temperature ionized plasma channel of the previous discharge does not completely dissipate, and the working fluid does not recover its insulating strength. Consequently, upon arrival of the next high-voltage pulse, arc discharges are more likely to occur, raising the local temperature and generating excess molten carbon fiber and SiC byproducts. Despite the increased concentration of heat, these adverse effects decrease the effective discharge rate and consequently the overall MRR. In conclusion, the optimum pulse frequency of ultrasonic vibration-assisted micro-EDM of C/SiC composites is about 120 kHz.

#### 3.2.4. The Effect of Pulse Width on MRR

Figure 7d shows the impact of pulse width on MRR with the parameters of a peak current of 30 Index, peak voltage of 100 V, pulse frequency of 120 kHz, and ultrasonic amplitude of 5 µm. Over the range of 3–5 µs, the MRR gradually increases with pulse width. However, above 5 μs, the trend is reversed and the MRR starts to drop. Since pulse width controls the time of each discharge, a longer pulse enables heat to be transferred better to the C/SiC composite, in favor of melting and vaporization, hence improving the MRR. Nonetheless, if the pulse width becomes too long, the high discharge energy results in larger erosion debris, which is not easily removed. This hinders the evacuation of debris, destabilizes the gap of discharge, and increases the chances of abnormal discharges, thereby reducing the MRR. Hence, the optimal pulse width of ultrasonic vibration-assisted micro-EDM of C/SiC composites is 5 µm.

## 4. Optimization of Experimental Design Using RSM

### 4.1. Plackett–Burman Experimental Design

Following the single-factor experiments described in the previous chapter, the optimum ranges of electrical parameters influencing MRR were determined. To further optimize these parameters, in the present study, the response surface methodology (RSM) was used. Initially, a PB design was used to screen the effect of several factors efficiently using fewer experimental trials. This approach helps to determine what parameters have the most significant effects on the response variable [17,18]. In this study, the MRR was the response variable, and five factors—peak current (*X*_1_), peak voltage (*X*_2_), pulse frequency (*X*_3_), pulse width (*X*_4_), and ultrasonic amplitude (*X*_5_)—were investigated. Each factor was tested at two levels (high and low), and the detailed experimental design is shown in Table 4 (N = 12).

The response values of MRR obtained by experiments are shown in Table 5.

The results obtained from the PB experimental design were fitted to a first-order linear model, expressed as follows:

(4) $$Y\;=\;\beta_{0}\;+\;\sum \beta_{i}X_{i}$$

In this equation, *Y* is the predicted objective function, *β*_0_ is the model intercept, *β*_i_ is the regression coefficient, and *X_i_* is the independent variable. The best regression equation of MRR (*Y*), which is obtained via multiple regression analysis of the experimental data given in Table 4, is as follows:

(5) $$Y=5.49-0.5908X_{1}-0.2008X_{2}+0.2958X_{3}-0.1875X_{4}+0.9008X_{5}$$

According to the *p*-values given in Table 6, the effect of the electrical parameters on the MRR is in the order of ultrasonic amplitude (*X*_5_) > peak current (*X*_1_) > pulse frequency (*X*_3_) > peak voltage (*X*_2_) > pulse width (*X*_4_).

### 4.2. The Steepest Ascent Test

The steepest ascent test is an extension of the results of the PB design, which proceeds along the steepest ascent path to find the direction of maximum response increase and the optimal range of factors that influence the response [19]. From the PB analysis, ultrasonic amplitude (*X*_1_), peak current (*X*_2_), and pulse frequency (*X*_3_) have significant effects on the MRR (*Y*). Among these factors, ultrasonic amplitude and pulse frequency have a positive effect on the MRR, whereas peak current has a negative effect. The design and results of the ascent test are summarized in Table 7.

The experimental results show that the MRR first increases and then decreases. When the ultrasonic amplitude is 5 μm, the peak current is 30 Index, and the pulse frequency is 120 kHz, the MRR reaches its maximum value. Therefore, these parameter settings were chosen as the center point for the next response surface optimization.

### 4.3. Box–Behnken Experimental Design

Based on the results of the PB experiment, the influence of the electrical parameters on the MRR was determined. In this section, the three most significant factors determined, ultrasonic amplitude, peak current, and pulse frequency, are further analyzed using a Box–Behnken design to determine their optimum level. The experimental data were analyzed using RSM and are presented in terms of a second-order regression equation.

(6) $$Y=\beta_{0}+\sum \beta_{i}X_{i}+\sum \beta_{ii}X_{i}^{2}+\sum \beta_{ij}X_{i}X_{j}$$

In the formula, Y is the predicted response; *β*_0_ is the intercept term; *β_i_* is the linear effect; *β*_ii_ is the quadratic effect; *β*_ij_ is the interaction effect; and *Xi*, *Xj* are independent variables. The influence of ultrasonic amplitude, peak current, and pulse frequency on the MRR is the most significant. After the steepest ascent test, the response surface analysis was performed, which was based on an ultrasonic amplitude of 5 µm, peak current of 30 Index, and pulse frequency of 120 kHz, in order to optimize the MRR. Table 8 shows the coded levels of each factor in the Box–Behnken design, and Table 9 shows the results of the response surface analysis.

We performed multiple regression fitting on the experimental data in Table 8 to obtain the quadratic multiple regression equation for MRR(*Y*):

(7) *Y* = 7.96 + 0.8112*X*_1_ + 0.7087*X*_2_ − 0.1650*X*_3_ − 0.2550*X*_1_*X*_2_ + 0.0575*X*_1_*X*_3_ − 0.9125*X*_2_*X*_3_ − 1.48*X*_1_^2^ − 1.09*X*_2_^2^ − 1.38*X*_3_^2^

The model’s coefficient of determination (R^2^) is 0.9212, which shows that the model fit is good between the predicted and experimental values. The adequate precision ratio of 7.4248 indicates that the model has a good signal-to-noise ratio. Furthermore, the Model F-value (Table 10) is 6.50, which indicates that the model is statistically significant.

**Figure 8:**
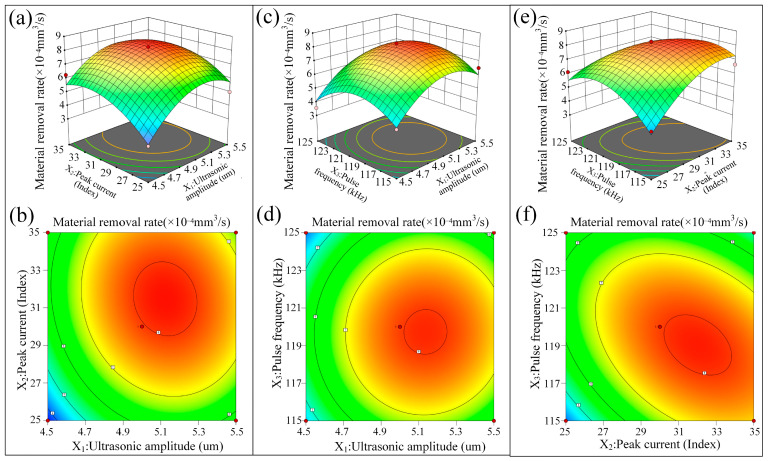
Response surface and contour plots of ultrasonic amplitude, peak current, and pulse frequency on MRR. (**a**) Response surface plot of ultrasonic amplitude and peak current. (**b**) Contour plot of ultrasonic amplitude and peak current. (**c**) Response surface plot of ultrasonic amplitude and pulse frequency. (**d**) Contour plot of ultrasonic amplitude and pulse frequency. (**e**) Response surface plot of peak current and pulse frequency. (**f**) Contour plot of peak current and pulse frequency.

The regression equation was analyzed to produce response surface and contour maps to show the effects of electrical parameters on the MRR, as shown in Figure 8. The shapes of the contour maps represent the strength of interactions between the three important factors: elliptical contours represent strong interactions and circular contours represent weak interactions. In the contour plots, warmer (red) colors indicate high MRR values. Figure 8a,b show the effects of ultrasonic amplitude (*X*_1_) and peak current (*X*_2_) on the MRR. The response surface in Figure 8a shows a unimodal distribution, with the MRR reaching its maximum near an ultrasonic amplitude of 5.1 µm and a peak current of 31.5 Index. The elliptical contour shape in Figure 8b suggests that there is a strong coupling between ultrasonic amplitude and peak current. Ultrasonic vibration improves the environment in the discharge gap and promotes the removal of debris. Concurrently, the higher peak current increases single-pulse discharge energy, which compensates for the limited energy contribution from ultrasonic vibration and, therefore, further enhances the MRR. Figure 8c,d show the combined effects of ultrasonic amplitude (*X*_1_) and pulse frequency (*X*_3_) on the MRR. The response surface in Figure 8c also indicates the unimodal trend, and the best condition is observed with an ultrasonic amplitude of 5.1 μm and a pulse frequency of 119 kHz, which gives the maximum MRR. The almost circular shape of the contour in Figure 8d indicates a weak interaction between these two parameters, which implies that the influence of these two parameters on MRR is almost additive rather than synergistic. Figure 8e,f show the effects of peak current (*X*_2_) and pulse frequency (*X*_3_) on the MRR. The response surface in Figure 8e has a fairly flat peak region, with the optimum occurring at about 31.5 Index and 119 kHz. The flattened elliptical contour in Figure 8f shows a moderate interaction between the peak current and the pulse frequency. As the peak current increases, the discharge energy per pulse increases, prolonging the time needed to evacuate debris from the discharge gap. In this case, increasing the pulse frequency increases the number of discharges per unit time, thus improving the MRR more effectively than changing either of the two factors separately.

The three sets of response surface analyses all converge on the same optimal parameter combination. The final optimized parameters for the maximum MRR are found as follows: peak current = 31.5 Index, peak voltage = 101 V, pulse frequency = 119 kHz, pulse width = 5 µs, and ultrasonic amplitude = 5.1 µm. These optimized values are highly consistent with the results of the single-factor experiments, which further confirms the validity of the model.

## 5. Conclusions

This study proposes an ultrasonic vibration-assisted micro-EDM method to solve the machining difficulties of C/SiC composites, which are difficult to overcome with conventional mechanical techniques. The following are the main conclusions:

(1) The experimental results demonstrate the feasibility of ultrasonic vibration-assisted micro-EDM for machining C/SiC composites. In single-factor experiments, the method enhanced the MRR of micro-holes to a much greater degree than conventional micro-EDM.
(2) The erosion mechanism of ultrasonic vibration-assisted micro-EDM for C/SiC composites mainly includes melting and vaporization of the SiC matrix, and the carbon fibers are removed by fragmentation and partial melting.
(3) The results of the PB experiment indicated that electrical parameters have the following influence order on the MRR: ultrasonic amplitude > peak current > pulse frequency > peak voltage > pulse width. On the basis of these results, a first-order regression model for MRR was developed to characterize the relationship between the process parameters and machining performance.
(4) Through the above single-objective optimization experiment, the following optimal parameters are obtained. The optimal parameter combination is the parameter combination with the maximum MRR under the combined action of five parameters. The optimal process parameters for ultrasonic vibration-assisted micro-EDM of C/SiC composites were determined as follows: peak current = 31.5 Index, peak voltage = 101 V, pulse frequency = 119 kHz, ultrasonic amplitude = 5.1 μm, and pulse width = 5 μs. Based on the Box–Behnken results, a quadratic polynomial regression equation for the MRR was derived through multiple regression analysis. The five parameters in this conclusion are the same as the experimental results of the optimized parameters. This set of parameters is the optimal parameter combination of a single-objective optimization target under the premise of ensuring the maximum MRR.

## Figures and Tables

**Table 1:** Physical properties of C/SiC composites.

Physical Properties	
Density (kg/m^3^)	2100
Melting point (°C)	2730
Poisson’s ratio	0.15–0.25
Specific heat capacity (J/g °C)	0.7–1.2
Thermal conductivity (W/m·K)	5–6.3
Tensile strength (MPa)	150
Flexural strength (MPa)	421
Fracture toughness (MPa·m^1/2^)	17.3
Elastic modulus (GPa)	74.42

**Table 2:** Processing parameters for single-factor experiments.

Process Parameters	Variable Values
Peak current (Index)	20, 25, 30, 35, 40
Peak voltage (V)	80, 90, 100, 110, 120
Pulse frequency (kHz)	100, 110, 120, 130, 140
Pulse width (μs)	3, 4, 5, 6, 7
Ultrasonic amplitude (μm)	0, 3, 4, 5, 6

**Table 3:** Physical properties of working fluid.

Dielectric Constant	Insulation Strength (mv/m)	Flash Point (°C)	Density (kg/m^3^)	Stickiness (mm^2^/s)	Combustion Point (°C)
3	14.22	134	813	7.0	243

**Table 4:** Plackett–Burman test design factors and levels.

Factor	Level	
Peak current (Index)	30	40
Peak voltage (V)	90	110
Pulse frequency (kHz)	110	130
Pulse width (μs)	4	6
Ultrasonic amplitude (μm)	3	5

**Table 5:** Plackett–Burman test design and response values.

No.	X_1_	X_2_	X_3_	X_4_	X_5_	Y
1	30	90	110	4	3	5.38
2	40	90	110	4	5	6.54
3	40	110	110	6	5	5.28
4	30	110	130	6	3	5.79
5	40	90	130	6	3	3.92
6	30	110	110	6	5	5.64
7	30	90	130	4	5	7.12
8	30	90	110	6	3	4.98
9	30	110	130	4	5	7.56
10	40	110	110	4	3	3.33
11	40	110	130	4	3	4.12
12	40	90	130	6	5	6.19

**Table 6:** Effects of various factors on MRR in Plackett–Burman experiment.

Y	Coefficient	F-Value	*p*-Value
	5.49	9.13	0.0090
X_1_	−0.5908	12.04	0.0133
X_2_	−0.2008	1.39	0.2828
X_3_	0.2958	3.02	0.1330
X_4_	−0.1875	1.21	0.3130
X_5_	0.9008	27.99	0.0018

**Table 7:** Design and results of steepest ascent test for MRR.

MRR	X_1_	X_2_	X_3_	Y
1	3.5	36	105	4.38
2	4	34	110	5.27
3	4.5	32	115	6.36
4	5	30	120	7.92
5	5.5	28	125	7.01
6	6	26	130	5.45

**Table 8:** Box–Behnken design of factor-level encoding for MRR.

MRR	Factor		Level	
Y		−1	0	1
X_1_	Ultrasonic amplitude (μm)	4.5	5	5.5
X_2_	Peak current (Index)	25	30	35
X_3_	Pulse frequency (kHz)	115	120	125

**Table 9:** Box–Behnken experimental design and results for MRR.

MRR	X_1_	X_2_	X_3_	Y
1	5	30	120	7.89
2	5	25	115	4.25
3	5	35	115	6.67
4	5.5	30	125	5.92
5	5.5	30	115	6.52
6	5	35	125	4.90
7	4.5	30	125	3.56
8	5.5	35	120	6.75
9	5	30	120	7.73
10	4.5	25	120	3.51
11	5	30	120	8.27
12	5	25	125	6.13
13	4.5	35	120	6.26
14	5.5	25	120	5.02
15	4.5	30	115	4.39

**Table 10:** Effects of various factors on MRR in Box–Behnken experiment.

	Coefficient	F-Value
Model	7.96	6.50
X_1_	0.8112	10.21
X_2_	0.7087	7.79
X_3_	−0.1650	0.4224
X_1_X_2_	−0.2550	0.5044
X_1_X_3_	0.0575	0.0256
X_2_X_3_	−0.9125	6.46
X_1_^2^	−1.48	15.77
X_2_^2^	−1.09	8.57
X_3_^2^	−1.38	13.67

## Data Availability

The original contributions presented in this study are included in the article. Further inquiries can be directed to the corresponding authors.

## Abbreviations

The following abbreviations are used in this manuscript:

EDM	Electric discharge machining
MRR	Material removal rate
RSM	Response surface methodology
CLSM	Confocal laser scanning microscope
SEM	Scanning electron microscope
PB	Plackett–Burman
2D	Two-dimensional
3D	Three-dimensional

## References

[1] Du J., Zhang H., Geng Y., Ming W., He W., Ma J., Cao Y., Li X., Liu K. (2019). A review on machining of carbon fiber reinforced ceramic matrix composites. Ceram. Int..

[2] Kumar S., Bablu M., Ranjan A., Manocha L.M., Prasad N.E. (2017). Fabrication of 2D C/C-SiC composites using PIP based hybrid process and investigation of mechanical properties degradation under cyclic heating. Ceram. Int..

[3] Liu C., Zhang X., Gao L., Jiang X., Wang X., Yang T. (2021). Study on damage characteristics and ablation mechanism in fiber laser trepan drilling of 2.5D C_f_/SiC composites. Int. J. Adv. Manuf. Technol..

[4] Pachaury Y., Tandon P. (2017). An overview of electric discharge machining of ceramics and ceramic based composites. J. Manuf. Process..

[5] Zhang Y.F., Chen W.W., Cheng H.W., Zhang Y.P. (2018). Machinability for C/SiC Composite Material by Electrical Discharge Machining. Mater. Sci. Forum.

[6] Guu Y.H., Hocheng H., Tai N.H., Liu S.Y. (2001). Effect of electrical discharge machining on the characteristics of carbon fiber reinforced carbon composites. J. Mater. Sci..

[7] Yue X., Li Q., Yang X. (2020). Influence of thermal stress on material removal of C_f__SiC composite in EDM. Ceram. Int..

[8] He W., He S., Du J., Ming W., Ma J., Cao Y., Li X. (2019). Fiber orientations effect on process performance for wire cut electrical discharge machining (WEDM) of 2D C/SiC composite. Int. J. Adv. Manuf. Technol..

[9] Ming W., Jia H., Zhang H., Zhang Z., Liu K., Du J., Shen F., Zhang G. (2020). A comprehensive review of electric discharge machining of advanced ceramics. Ceram. Int..

[10] Sachidhananda T.G., Chandrashekhar V.A. (2021). Electric Discharge Machining of Conducting Ceramics—A Review. Mater. Sci. Forum.

[11] Wang Y., Fan L., Shi J., Dong Y., Fu Z. (2023). Effect of cavitation on surface formation mechanism of ultrasonic vibration-assisted EDM. Int. J. Adv. Manuf. Technol..

[12] Zhang P., Yin Z., Yu M., Tao D., Yu D., Zhang Q., Li H. (2024). Investigating mechanisms of debris removal in ultrasonic vibration-assisted EDM drilling. Int. J. Mech. Sci..

[13] Xing Q., Yao Z., Zhang Q. (2021). Effects of processing parameters on processing performances of ultrasonic vibration-assisted micro-EDM. Int. J. Adv. Manuf. Technol..

[14] Wang Y., Liu Z., Shi J., Dong Y., Yang S., Zhang X., Lin B. (2020). Analysis of material removal and surface generation mechanism of ultrasonic vibration-assisted EDM. Int. J. Adv. Manuf. Technol..

[15] Qu S., Gong Y., Yang Y., Cai M., Sun Y. (2018). Surface topography and roughness of silicon carbide ceramic matrix composites. Ceram. Int..

[16] Liu Y., Liu Z., Wang X., Huang T. (2020). Experimental study on tool wear in ultrasonic vibration–assisted milling of C/SiC composites. Int. J. Adv. Manuf. Technol..

[17] Ganapathy S., Palanivendhan M., Balasubramanian P., Suresh M. Process parameter optimization on EN8 steel in Electric Discharge Machining (EDM) using Response Surface Methodology (RSM) Technique. Proceedings of the IOP Conference Series Materials Science and Engineering.

[18] Tzeng C.J., Chen R.Y. (2013). Optimization of electric discharge machining process using the response surface methodology and genetic algorithm approach. Int. J. Precis. Eng. Manuf..

[19] Jensen W.A. (2017). Response Surface Methodology: Process and Product Optimization Using Designed Experiments 4th edition. J. Qual. Technol..

